# Pseudoaneurysm of a branch of the femoral circumflex artery as a complication of revision arthroscopic release of the iliopsoas tendon

**DOI:** 10.1051/sicotj/2017012

**Published:** 2017-03-22

**Authors:** Naoki Nakano, Laughter Lisenda, Vikas Khanduja

**Affiliations:** 1 Department of Trauma and Orthopaedics, Addenbrooke’s Hospital, Cambridge University Hospitals NHS Foundation Trust Hills Road Cambridge CB2 0QQ UK

**Keywords:** Pseudoaneurysm, Femoral circumflex artery, Arthroscopic iliopsoas tendon release, Iliopsoas snapping, Embolisation

## Abstract

Treatment of painful internal snapping hip via arthroscopic release of the iliopsoas tendon is becoming the preferred option over open techniques because of the benefits of minimal dissection and fewer complications. However, complications do occur with arthroscopic techniques as well. We present the case of a 33-year-old woman who presented with painful internal snapping of her right hip and underwent arthroscopic release of the iliopsoas tendon. Following the procedure she continued to complain of pain in her groin and was therefore investigated further with a magnetic resonance imaging (MRI) which revealed a swelling near the femoral circumflex vessels. A computed tomography (CT) angiogram revealed a 15 mm pseudoaneurysm of the femoral circumflex artery, which was successfully treated by selective catheterisation and embolisation. Hip arthroscopists should be sufficiently familiar with the vascular anatomy around the hip and keep this complication in mind when releasing the iliopsoas tendon arthroscopically especially in revision cases with adhesions.

## Introduction

Internal snapping of the hip occurs when the iliopsoas tendon snaps over the iliopectineal eminence or across the femoral head as the hip is brought from flexion, abduction and external rotation to extension. If non-operative treatment, which includes physiotherapy to stretch the muscle, fails then open or arthroscopic release of the iliopsoas tendon has been described as being effective in relieving the snapping sensation, reducing pain and restoring function [1]. Complications from open procedures such as transient sensory loss in the anterolateral aspect of the thigh have been reported to occur with high frequency [2], thus, arthroscopic release of the iliopsoas tendon was introduced as an adjunct to hip arthroscopy for operative treatment of this problem [1, 3]. Arthroscopic release of the iliopsoas consists of dividing the tendinous portion of the iliopsoas between the level of the acetabulum and the lesser trochanter, which leads to functional lengthening of the musculotendinous unit while leaving the muscle belly intact, and it has been shown to effectively alleviate pain associated with internal snapping of the hip [1, 2].

Except for persistent snapping after release, complications following arthroscopic release of the iliopsoas tendon have not been reported thus far [4]. In this report, we present a rare case of a pseudoaneurysm, which occurred in the femoral circumflex artery after arthroscopic release of the iliopsoas tendon.

## Case presentation

A 33-year-old woman was referred to our tertiary young adult hip service for evaluation of right hip pain, which had been affecting her for over six months along with mechanical symptoms of snapping. She had undergone an arthroscopy of her hip previously for an acetabular labral tear and recovered well following the same. She did not report of any definite history of injury, which had led to the onset of her symptoms.

The examination of her right hip revealed that she had a normal gait, and flexion was limited to 90°. Internal rotation in 90°of flexion was limited to 20° and external rotation in 90°of flexion was 40°. The impingement test was positive and there was definite snapping palpable in the groin on extending her hip from a flexed and abducted position, which was painful. Dorsalis pedis was well palpable. Plain radiographs did not reveal any signs of dysplasia and showed a well-preserved joint space. A dynamic ultrasound scan showed that the iliopsoas tendon snapped as she extended her hip from flexed and externally rotated position.

Physiotherapy to stretch her iliopsoas and an ultrasound guided injection of steroid was not quite successful and since she was quite symptomatic, revision arthroscopic intervention was planned. At arthroscopy, the labrum was frayed and therefore debrided but was stable and the iliopsoas was released from the peripheral compartment via the transcapsular approach. Whilst dissecting the iliopsoas tendon, a gush of blood was found, and the oozing channel was coagulated with the radiofrequency probe. After 20 min of the pressure dressing, there was no further ooze found. There was also no evidence of any distal vascular deficit, and the Doppler showed a good pulse at the dorsalis pedis and the posterior tibial artery.

**Figure 1.**
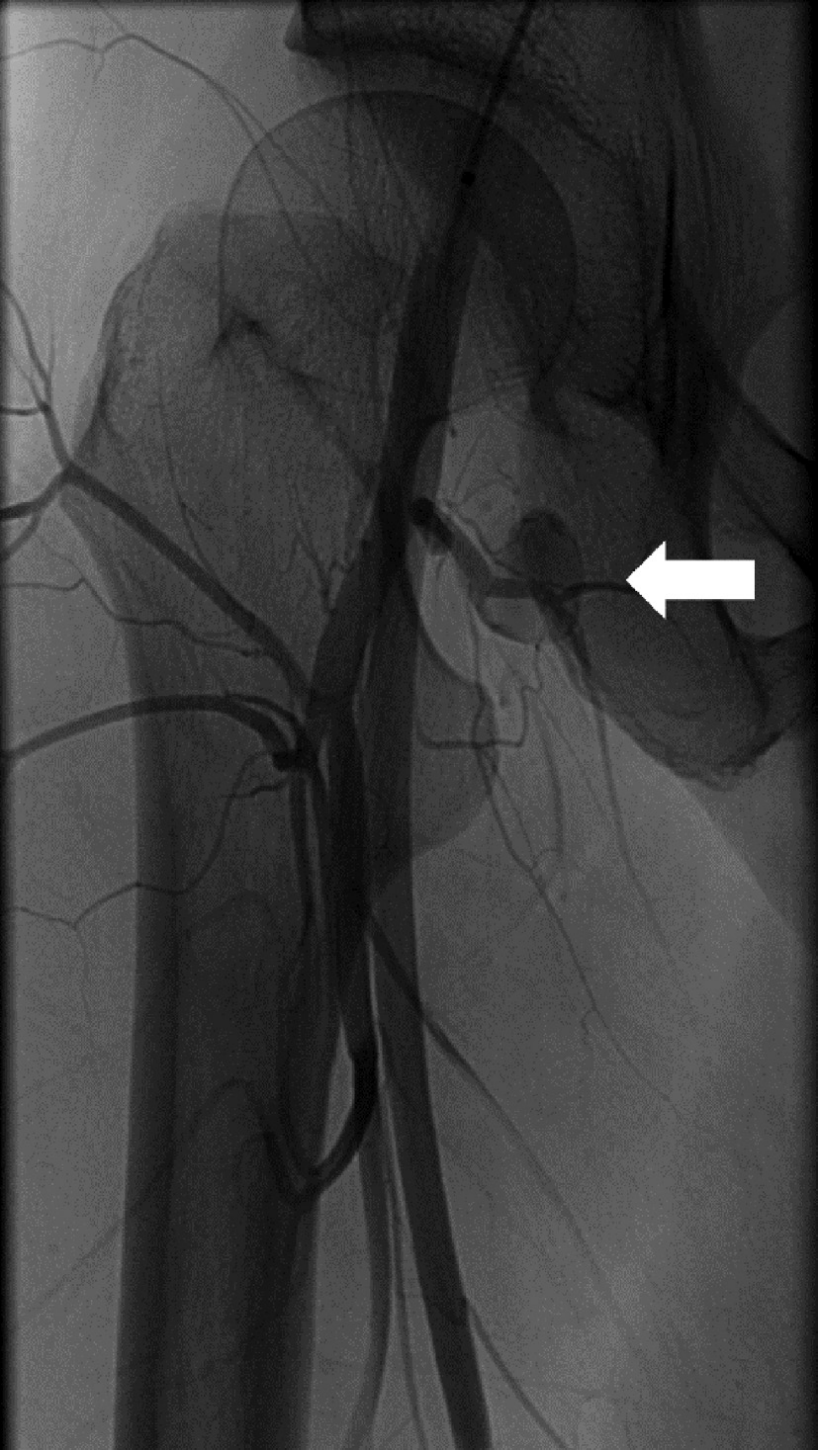
An angiogram of pre-embolisation. A 15 mm pseudoaneurysm which arose from a circumflex femoral branch that arose directly from the posterior aspect of the femoral circumflex artery is shown (arrow).

**Figure 2.**
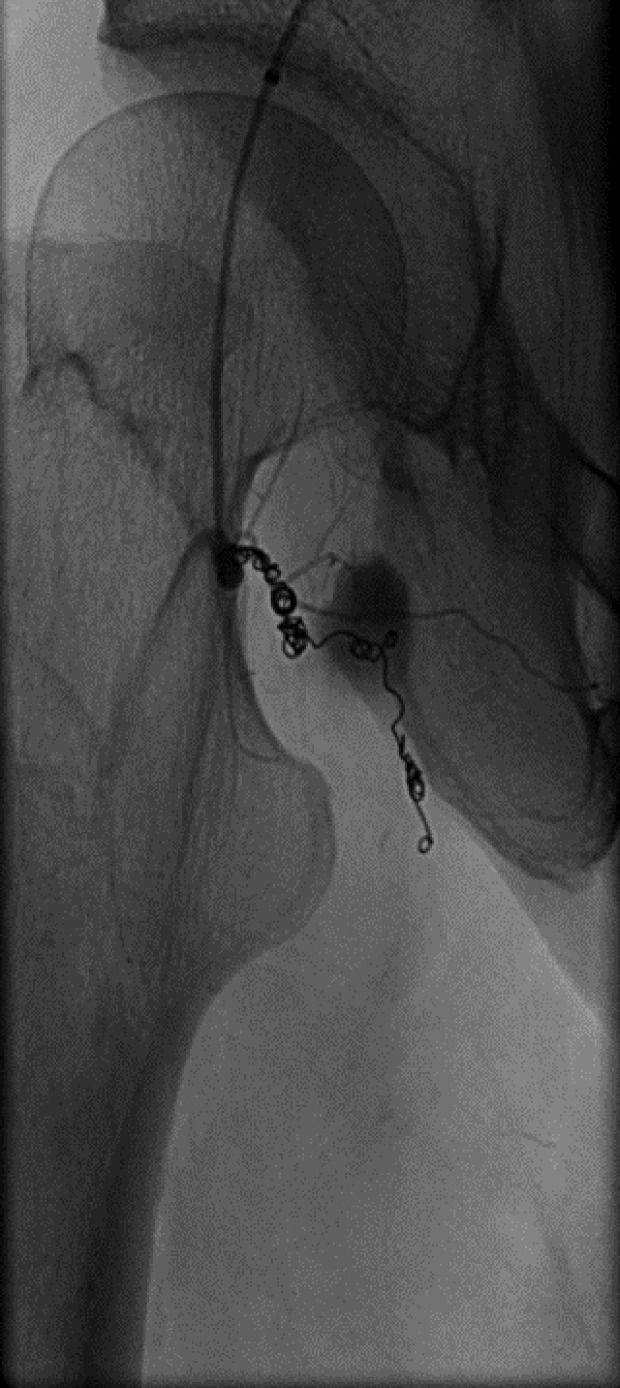
An angiogram of post-embolisation. A pseudoaneurysm was embolised with micro coils.

**Figure 3.**
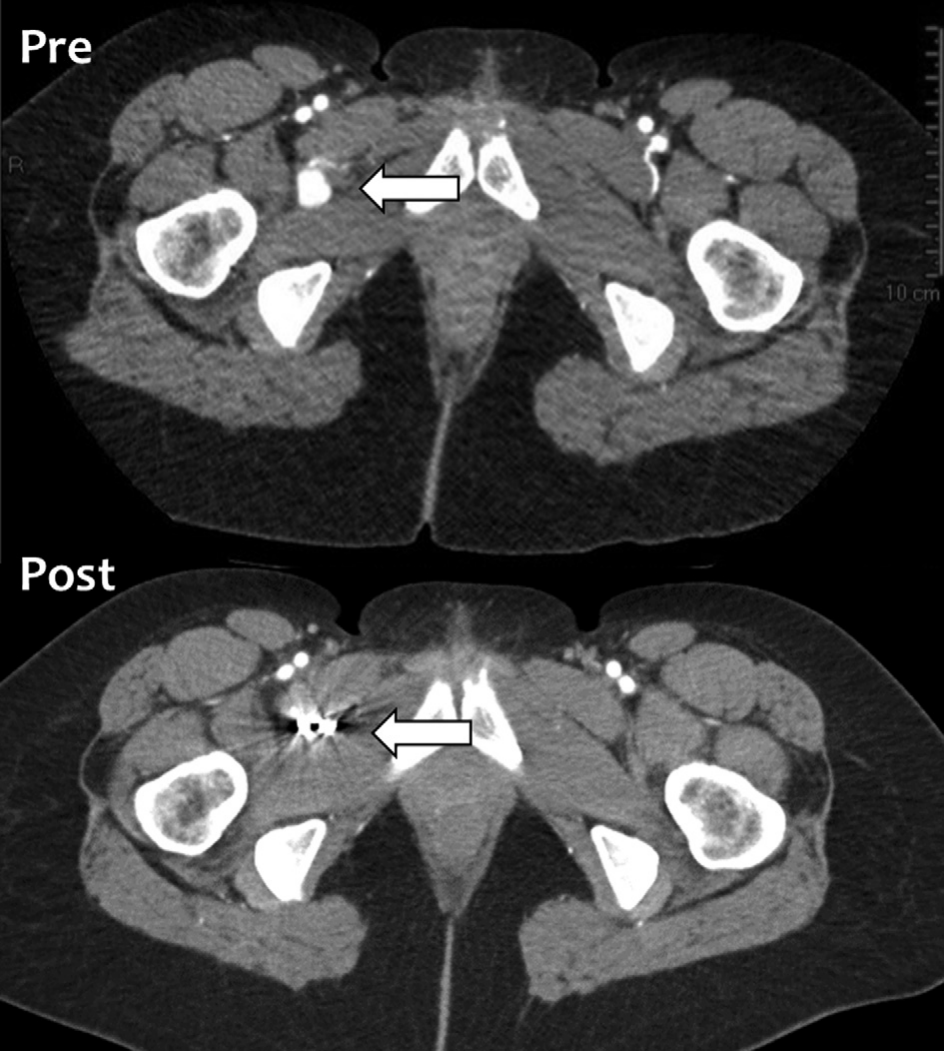
A CT appearance of pre- and post-embolisation. A pseudoaneurysm was embolised with micro coils (arrow).

However, two weeks following the procedure, she still had an uncomfortable feeling in her right hip. A magnetic resonance imaging (MRI) scan was arranged which showed a 20 mm reasonably well-defined focus of abnormal signal immediately medial to the psoas tendon just proximal to its insertion, which was consistent with a haematoma. An angiogram showed that there was a 15 mm pseudoaneurysm in the right groin, which arose from a circumflex femoral branch that arose directly from the posterior aspect of the femoral circumflex artery, immediately proximal to the profunda bifurcation (Figure 1). She underwent selective catheterisation and embolisation with micro coils (Figure 2). The aneurysm was accessed from the left common femoral artery by retrograde puncture, and 4Fr sheath was used. Following embolisation, she was asymptomatic and a repeat X-ray, computed tomography (CT) (Figure 3) and MRI showed satisfactory embolisation of the pseudoaneurysm with preservation of perfusion of the surrounding muscles.

## Discussion

We present a rare case of a pseudoaneurysm following arthroscopic release of the iliopsoas tendon for painful iliopsoas snapping. Although, arthroscopy has been thought of as a relatively safe procedure, it is not free of complications [5–11]. An endoscopic release of the iliopsoas tendon can be done at the level of the hip capsule via the central or peripheral compartments or at its attachment at the lesser trochanter [3]. The posterior surface of the iliopsoas muscle and tendon is in close contact with the anterior capsule of the hip. This close relationship allows performing an iliopsoas tenotomy from the peripheral compartment of the hip.

**Figure 4.**
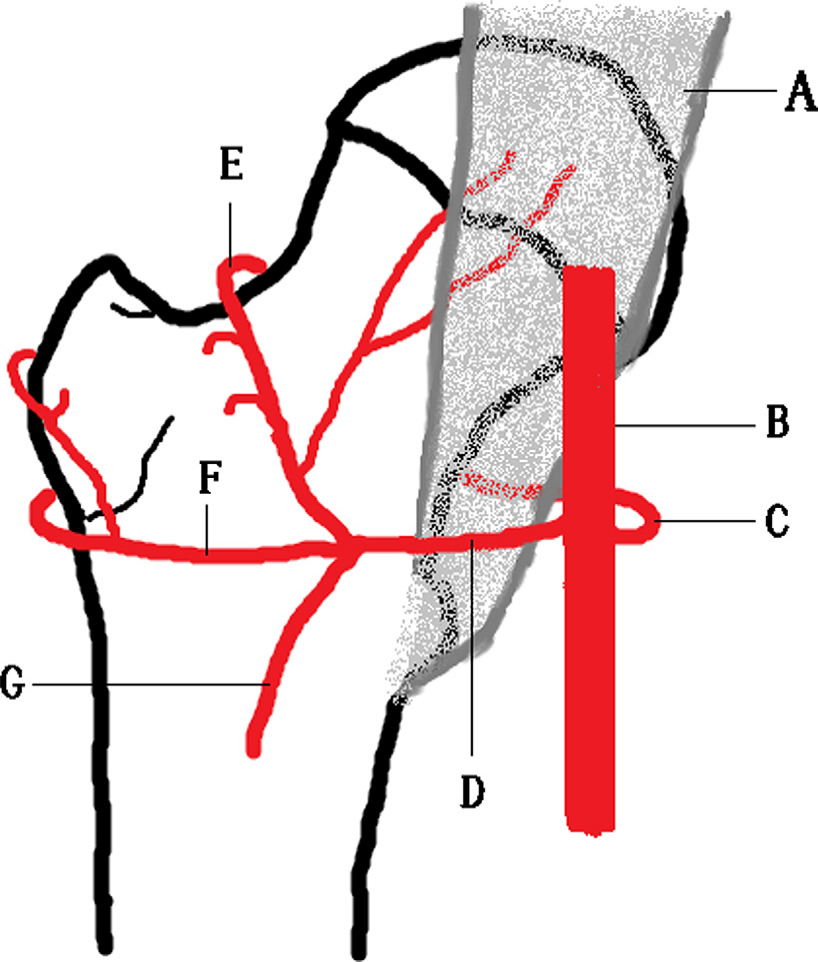
Arteries around the proximal femur. (A) Iliopsoas muscle, (B) profunda femoris artery, (C) medial femoral circumflex artery, (D) lateral femoral circumflex artery, (E) ascending branch of lateral femoral circumflex artery, (F) transverse branch of lateral femoral circumflex artery, (G) descending branch of lateral femoral circumflex artery.

The femoral circumflex artery consists of two branches: medial femoral circumflex artery (MFCA) and lateral femoral circumflex artery (LFCA). The MFCA has its origin from the deep femoral artery of the femoral artery [12]. The MFCA usually has five branches: ascending, descending, acetabular, superficial, and deep. The deep branch of the MFCA is the one which is the most responsible for the vascularisation of the femoral head and neck. It has its origin medially from the femoral artery between the pectineus and iliopsoas tendons, along the inferior border of externus obturator muscle. The LFCA arises from the lateral side of the deep femoral artery in most of the cases. From this point, it goes to a lateral position just behind the sartorius and rectus femoris and between the divisions of the femoral nerve. At this level, it gives origin to the ascending, transverse and descending branches (Figure 4).

In this current case, adhesions around the hip joint due to the previous arthroscopy could have contributed to the tethering of the vessels around the zona orbicularis leading to the formation of the pseudoaneurysm. It should also be noted that a vessel is not the only structure which could be damaged during arthroscopic release of the iliopsoas tendon. Some branches of the femoral nerve lie directly over the anterior surface of the iliopsoas muscle. These branches are at risk when a release of the iliopsoas tendon is performed, although, in a more distal position, they are protected by the presence of the iliopsoas bursa and the vastus intermedius [3]. Hip arthroscopists should bear the possibility of adhesions and these complications especially when performing revision hip arthroscopy; however, it is not difficult to comprehend that such a complication could happen even during an open procedure.

As a conclusion, we present a rare case of a pseudoaneurysm, which occurred in the femoral circumflex artery after revision arthroscopic release of the iliopsoas tendon. Hip arthroscopists should bear this complication in mind when releasing the snapping iliopsoas tendon arthroscopically especially in the background of adhesions in revision cases.

## Conflict of interest

NN, LL and VK certify that they have no financial conflict of interest in connection with this article.
